# PSCA and Oct-4 Expression in the Benign and Malignant Lesions of Gallbladder: Implication for Carcinogenesis, Progression, and Prognosis of Gallbladder Adenocarcinoma

**DOI:** 10.1155/2013/648420

**Published:** 2013-08-01

**Authors:** Qiong Zou, Leping Yang, Zhulin Yang, Jiangsheng Huang, Xi Fu

**Affiliations:** ^1^Department of Pathology, Third Xiangya Hospital, Central South University, Changsha, Hunan 410013, China; ^2^Research Laboratory of Hepatobiliary Diseases, Second Xiangya Hospital, Central South University, Changsha, Hunan 410011, China

## Abstract

PSCA and Oct-4 have been thought as markers of cancer stem cells. Although overexpression of PSCA and Oct-4 in cancer has been reported, little is known about the clinical and pathological significance with PSCA and Oct-4 expression in gallbladder adenocarcinoma. In this study, overexpression of PSCA and Oct-4 was detected in gallbladder adenocarcinoma (54.6% and 55.6%). Less expression of PSCA and Oct-4 was detected in the pericancerous tissues (19.6% and 21.7%), gallbladder polyps (13.3% and 13.3%), and gallbladder epithelium with chronic cholecystitis (14.3% and 14.3%). The overexpression of PSCA and Oct-4 was significantly associated with differentiation, tumor mass, lymph node metastasis, invasion of gallbladder adenocarcinoma, and decreased overall survival. Our study suggested that overexpression of PSCA and Oct-4 might be closely related to the carcinogenesis, progression, metastasis, or invasive potential and prognosis of gallbladder carcinoma.

## 1. Introduction

Increasing data support that cancer is a stem cell-based disease. Cancer stem cells (CSCs) are a subpopulation of tumor cells that selectively possess tumor initiation, self-renewal capacity, and ability to give rise to bulk populations of nontumorigenic cancer cell progeny through differentiation. CSCs have been found in different human cancers, including prostate cancer [1], breast cancer [2], colon cancer [3], pancreatic cancer [4], and head and neck squamous cell carcinoma [5]. These observations have dramatic biological and clinical significance due to the increasing evidence suggesting that recurrence of human tumor and treatment failure may reflect the intrinsic quiescence and drug resistance of CSCs. 

The prostate stem cell antigen (PSCA) gene was originally identified through an analysis of genes upregulated in the human prostate cancer LAPC-4 xenograft model [6]. The PSCA gene is located on chromosome 8q24.2 and encodes a 123 amino acid cell-surface protein with 30% homology to stem cell antigen type2 (SCA-2), an immature lymphocyte surface marker. In some studies, PSCA protein is highly expressed by a large percentage of human prostate tumors, including metastatic and hormone-refractory cancers, but it has limited expression in normal tissues [7–9]. Elevated level of PSCA in prostate tumor is correlated with the increased Gleason score, advanced stage, progression, and death. PSCA may therefore be a useful predictor of tumor biology and a useful target of immunotherapy against prostate cancer [7–9]. Moreover, PSCA is also strongly expressed in nonprostatic malignancies, including bladder cancer [10, 11], pancreatic cancer [12–14], renal cell carcinoma [15], and diffuse-type gastric cancer [16]. The function of PSCA in tumor biology and the regulatory mechanism of PSCA expression remain unknown yet. Previous studies have revealed the activities of some homologues of PSCA including CD177, CD59, and uPAR belonging to Ly-6 gene superfamily [17–20]. Their activities comprise cell adhesion, cell migration, and inhibition of complement-mediated hemolysis [17, 18]. Gu et al. speculated that c-myc amplification might associate with PSCA overexpression in prostate cancer [8]. C-myc is known to be one of the important oncogenic proteins. However, PSCA expression in the benign and malignant lesions of the gallbladder has not yet been identified.

The Oct-4 gene, a POU- (Pit-Oct-Unc-) domain octamer-binding transcription factor, is a key regulator of self-renewal in embryonic stem cells [21, 22]. Oct-4 appears to function in maintaining a pluripotent potential in embryonic stem cells and embryonic germ cells [21, 22], and it has been shown to be necessary for embryonic stem cell formation and self-renewal [23, 24]. It has been reported that Oct-4 was expressed in human tumors but not in normal somatic tissues [25]. Under the hypothesis of “cancer stem cell” in somatic tumors, an increasing number of researchers explored the expression of Oct-4 in human somatic tumors and somatic tumor cell lines. Some studies have shown that Oct-4 gene was expressed in human breast cancer cells [26], lung cancer cells [27], bladder transitional cell carcinoma samples and cell lines [28], prostate cancer [29], and oral cancer [30]. The expression of Oct-4 has also been shown in cancer stem cell-like cells (CSCLCs), including human breast CSCLCs [31]. Overexpression of Oct-4 was potentially correlated with tumorigenesis and can affect some tumor behaviors, such as tumor metastasis and recurrence or resistance to therapies [32]. It is possible that overexpression of Oct-4 may contribute to the neoplastic process and play a role in cancer stem cell theory [33, 34]. Although Oct-4 has been thought to be a molecular marker of tumor germ cells, little is known about its expression in gallbladder carcinoma (GBC).

GBC is a relatively rare neoplasm but highly lethal disease despite the improvement in diagnostic techniques. With an incidence of 0.8% to 1.2%, this is the most common biliary tract tumor and the fifth most common malignancy of the gastrointestinal tract [35]. The 5-year prognosis for all stages of gallbladder carcinoma is about 5% [36, 37], while the median survival for patients with suspected cancers is 9.2 months [38]. However, the molecular mechanisms involved in the pathogenesis of GBC have not been clearly defined; especially the CSC and CSC antigens underlining gallbladder carcinogenesis are poorly understood.

Because of the lack of patients with bile duct neoplasm, little is known about the clinical or pathological significance with PSCA and Oct-4 expression in gallbladder adenocarcinoma. In this study, we used immunohistochemical analysis to examine PSCA and Oct-4 expression in the benign and malignant lesions of the gallbladder and attempted to elucidate the clinical and pathological significance of changes in GBC.

## 2. Materials and Methods

### 2.1. Specimens and Clinicopathologic Materials

A total of 204 gallbladder specimens, including 108 gallbladder adenocarcinoma tissues, 46 peritumoral tissues, 15 gallbladder polyps, and 35 chronic cholecystitis tissues, were studied with preapproval from Ethics Committee of Human Study of Central South University. This study complied with the Code of Ethics of the World Medical Association (Helsinki Declaration of 1964, as revised in 2002). These samples were collected between 1996 and 2006. All diagnoses were based on morphological criteria, immunohistochemical staining, and clinical findings. Among the 108 adenocarcinoma cases, 31 cases are males (28.7%), and 77 cases are females (71.3%) with an average age of 52.6 ± 11.2 years. The histopathologic types of these adenocarcinoma cases include 9 adenoma canceration (8.2%, 7 well-differentiated and 2 moderately differentiated), 29 well-differentiated adenocarcinoma (26.9%), 29 moderately differentiated adenocarcinomas (26.9%), 30 poorly differentiated adenocarcinoma (27.8%), and 11 mucinous adenocarcinomas (10.2%). The invasion was evaluated according to the standard criteria for T-stages [39]. Among the 108 adenocarcinomas, 14 cases are at T1, 35 cases are at T2, 37 cases are at T3, and 22 cases are at T4 stage; 59 cases had regional lymph node metastasis (54.6%), and 58 cases had gallstones (53.7%). Among these adenocarcinoma tissues, 34 cases were with radical resection (31.5%), 48 were with palliative surgery (44.4%), and no operations were performed for 26 cases (24.1%) with only surgery biopsy due to local invasion into critical structures or metastasis beyond regional confines. Survival information of 67 cases among the 108 adenocarcinomas was obtained through letters and phone calls. Among them, 20 cases survived over 1 year, and 47 cases survived less than 1 year. The chronic cholecystitis, gallbladder polyps, and peritumoral tissues were diagnosed according to the published standard criteria [40]. According to the criteria for dysplasia described by Dowling and Kelly [41], the 35 cases of chronic cholecystitis (15 had chronic cholecystitis only, and 20 had chronic cholecystitis accompanied by gallbladder stone) were classified into normal, mild, moderate, or severe dysplasia: 11 cases without cellular atypia as normal mucosa, 12 cases with mild cellular atypia as mild dysplasia, 7 cases with moderate cellular atypia as moderate dysplasia, and 5 cases with severe cellular atypia as severe dysplasia. Among the 15 cases with gallbladder polyps, pathological examination confirmed that 10 polyps had normal-to-mild epithelial dysplasia, and 5 had moderate to severe dysplasia. Among the 46 peritumoral tissues (distance from cancer ≥ 3 mm), 10 tissues were normal, 10 tissues were mild dysplastic, 12 tissues were moderate dysplastic, and 14 tissues were severe dysplastic. 

All of these specimens were fixed by 4% formaldehyde, followed by conventional paraffin-embedded sectioning.

### 2.2. Immunohistochemistry

The staining of PSCA and Oct-4 was made by immunohistochemical method of EnVision (ChemMateTMEnVison +/HRP/DAB; rabbit/mouse two step staining method) according to the manufacturer's protocol (DAKO laboratories, CA, USA). All tissues were cut into 4 *μ*m thick sections. The rabbit anti-human PSCA and Oct-4 antibodies were purchased from Maixin Biotechnology (Maixin, Inc., Fujian, China). The positive control of PSCA is prostate cancer section, and the positive control of Oct-4 is seminoma section. The negative control of PSCA and Oct-4 is the 5% fetal bovine serum liquid (pH7.4) substituted primary antibody. The positive product of PSCA and Oct-4 was brown-yellow, which mainly located in cytoplasm, occasionally in nucleus. The percentage of cells staining positive for PSCA and Oct-4 was estimated: <5%, (0 score); 6%–10% (1 score); 11%–20% (2 score); 21%–50% (3 score); >50%(4 score). The staining intensity was classified as negative (0 score), weak (1 score), moderate (2 score), or strong (3 score). Then, two parts of the score were added up to get the case total score. The cases with total score ≤2 were considered to be negative cases; the cases with total score ≥3 were considered to be positive cases [42, 43].

### 2.3. Statistical Analysis

Data was analyzed using the statistical package for the Social Sciences Version 13.0 (SPSS 13.0). The interrelationships of PSCA or Oct-4 expression with clinicopathological features were analyzed using *χ* ^2^ or Fisher's exact test. The Kaplan-Meier survival analysis and log-rank tests were used for univariate survival analysis. The Cox proportional hazards model was used for multivariate survival analysis and to determine the 95% confidence interval.

## 3. Results

### 3.1. Expression of PSCA and Oct-4 in the Benign and Malignant Lesions of Gallbladder

**Figure 1:**
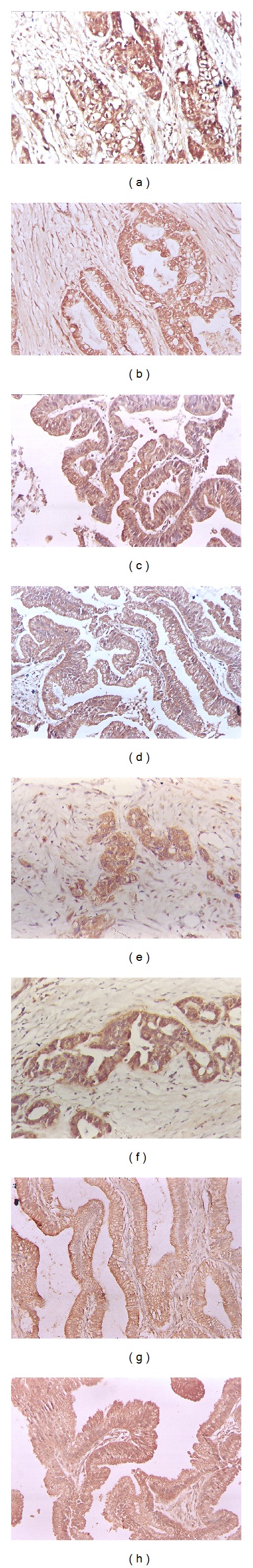
PSCA and Oct-4 expression in the benign and malignant lesions of gallbladder. EnVision immunohistochemistry, original magnification ×200. PSCA and Oct-4 positive reaction was mainly localized in the cytoplasm. (a) PSCA positive expression in poorly differentiated gallbladder adenocarcinoma; (b) PSCA positive expression in severe atypical hyperplasia of gallbladder epithelium of peritumoral tissues. (c) PSCA positive expression in adenoma with moderate dysplasia; (d) Oct-4 positive expression in moderately differentiated gallbladder adenocarcinoma; (e) Oct-4 positive expression in severe atypical hyperplasia of gallbladder epithelium of peritumoral tissues; (f) Oct-4 positive expression in moderate atypical hyperplasia of gallbladder epithelium in chronic cholecystitis; (g) Oct-4 positive staining with 4 scores is observed in adenomatous polyps with moderate atypical hyperplasia; (h) Oct-4 positive staining with 4 scores is observed in chronic cholecystitis with moderate atypical hyperplasia.

EnVision immunohistochemistry revealed that positive reaction for PSCA and Oct-4 was mainly localized in the cytoplasm, occasionally in the nucleus (Figure 1). As shown in Table 1, among the 108 cases of gallbladder adenocarcinoma, PSCA and Oct-4 were positively expressed in 59 (54.6%) and 60 (55.6%) cases, respectively. Among the 46 cases of pericancerous tissues, PSCA and Oct-4 were positively expressed in 9 (19.6%) and 10 (21.7%) cases, respectively. Among the 15 polyp cases, positive expression of both PSCA and Oct-4 was observed in 2 cases (13.3%). Among 35 cases of chronic cholecystitis, there were 5 positive cases for both PSCA and Oct-4 (14.3%). The rates of PSCA and Oct-4 positive expression in adenocarcinoma were significantly higher than the in the pericancerous tissues (*χ* ^2^ PSCA  =  16.08; *χ* ^2^ Oct-4 = 14.88;  *P* < 0.01), polyp (*χ* ^2^  PSCA = 8.99;  *χ* ^2^ Oct-4 = 9.39;  *P* < 0.01), and gallbladder epithelium with chronic cholecystitis (*χ* ^2^  PSCA = 17.40;  *χ* ^2^  Oct-4 = 18.16;  *P* < 0.01). Interestingly, moderate-to-severe atypical hyperplasia was observed in the epithelium of benign gallbladder with higher PSCA and Oct-4 expression. These results suggested that both PSCA and Oct-4 are highly expressed in gallbladder adenocarcinoma, and they could be regarded as molecular markers to evaluate the malignant transformation of GBC.

### 3.2. The Correlation between PSCA and Oct-4 Expression Levels with Clinicopathologic Features of Gallbladder Adenocarcinoma

As shown in Table 2, the positive rates of PSCA and Oct-4 were significantly lower in the cases of well-differentiation, with small tumor size (<2 cm in diameter), nometastasis of lymph node, and at T1 stage than in cases of poor differentiation, with larger tumor size (≥2 cm in diameter), with metastasis of lymph node, and at T3 or T4 stage (*P* < 0.05 or *P* < 0.01). There was no significant correlation between the expression levels of PSCA and Oct-4 with other clinicopathological characteristics, such as the sex, age, and history of gallstones. 

### 3.3. The Association of PSCA and Oct-4 Expression with Survival in Gallbladder Adenocarcinoma Patients

**Figure 2:**
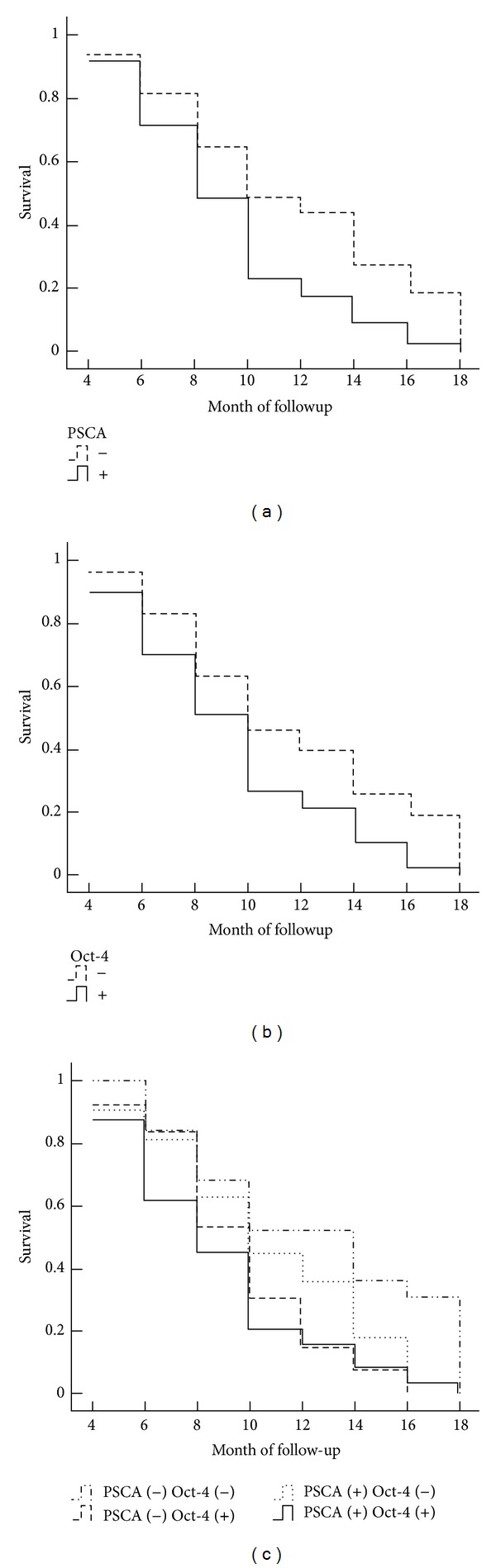
PSCA and/or Oct-4 expression and survival in patients with adenocarcinoma of gallbladder. (a) The Kaplan-Meier plots of overall survival in patients with gallbladder adenocarcinoma and with PSCA positive and negative expression Levels. (b) The Kaplan-Meier plots of overall survival in patients with gallbladder adenocarcinoma and with Oct-4 positive and negative expression Levels. (c) The Kaplan-Meier plots of overall survival in patients with gallbladder adenocarcinoma and with PSCA (+) (−) and Oct-4 (+) (−).

Survival information of 67 patients among the 108 gallbladder adenocarcinoma patients was followed up through letters and phone calls. Among the 67 cases, 35 (52.2%) and 37 (55.2%) patients were PSCA and Oct-4 positive, respectively. Of the 67 cases with followup, 20 cases survived over one year, and 47 cases survived less than one year, with a mean survival time of 9.6 ± 5.2 months. The positive rate of PSCA and Oct-4 in the cases that survived over one year was significantly lower than in those cases that survived less than one year (PSCA:  30.0%  versus  61.7%,  *P* < 0.05;  Oct-4:  35.0%  versus  63.8%, *P* < 0.05). The univariate Kaplan-Meier survival analysis revealed that tumor pathological type (*P* = 0.031), tumor diameter (*P* = 0.003), lymph node metastasis (*P* = 0.005),  T stages (*P* = 0.003), and operative procedure (*P* < 0.000) were associated with overall survival in cases with adenocarcinoma. The overall survival was inversely associated with negative or decreased expression of PSCA (*P* = 0.013) and Oct-4 (*P* = 0.029). In addition, the average survival time in patients having PSCA (−) Oct-4 (−) expression was significantly higher than in ones having PSCA (+) Oct-4 (+) (*P* = 0.013) (Table 3; Figure 2). The Cox multivariate survival analysis showed that the overall survival was negatively associated with tumor pathological type, tumor diameter, lymph node metastasis, T stages, and operative procedure as well as PSCA or Oct-4 expression. The above factors were independent prognosis markers for gallbladder carcinoma. According to the risk rank, PSCA overexpression was the most significant predicator of short overall survival, followed by lymph node metastasis (Table 4). 

## 4. Discussion

Although the overexpression of PSCA and Oct-4 was previously reported in some cancer, to our knowledge, this is the first report showing PSCA and Oct-4 expression in the benign and malignant lesions of the gallbladder. In this study, we used an extensive collection of human gallbladder adenocarcinoma and benign lesions of the gallbladder samples to demonstrate the clinical and pathological significance of PSCA and Oct-4 expression in GBC. We found that 54.6% of adenocarcinoma exhibited overexpression of PSCA and 55.6% of adenocarcinoma exhibited a significantly elevated Oct-4 level. In the gallbladder epithelium of benign lesions with higher PSCA and Oct-4 expression, moderate-to-severe atypical hyperplasia was observed. Moreover, PSCA and Oct-4 overexpression levels were important factors associated with the invasion, metastasis, and poor prognosis of gallbladder adenocarcinoma. Thus, overexpression of PSCA and/or Oct-4 can be used as a biological marker to predict the progression and prognosis of gallbladder adenocarcinoma. 

Atypical epithelial hyperplasia of the gallbladder is thought to be one of the premalignant conditions of gallbladder carcinoma [44]. It has been estimated that the time period required for progression from atypical hyperplasia, to carcinoma in situ, to invasive carcinoma would be about 15 years [45]. Severe atypical hyperplasia and carcinoma have been found in situ in more than 90% of GBCs [46]. In this study, we examined the PSCA and Oct-4 expression in the benign and malignant lesions of the gallbladder tissues. Our study showed that the rate of PSCA and Oct-4 positive expression was significantly higher in adenocarcinoma than that in pericancerous tissues, polyp, and gallbladder epithelium with chronic cholecystitis. Moderate-to-severe atypical hyperplasia was observed in the gallbladder epithelium of benign lesions with higher PSCA and Oct-4 expression. In contrast, in the epithelium of gallbladder with mild atypical hyperplasia or normal tissues, PSCA and Oct-4 expression was lower or negative. To determine the clinical significance of PSCA and Oct-4 expression, we analyzed the correlations of PSCA and Oct-4 expression levels with several clinicopathological factors. We found that PSCA and Oct-4 overexpression was significantly correlated with the differentiation of adenocarcinoma, metastasis of lymph node, and T stages. Thus, PSCA and Oct-4 might play a critical role in carcinogenesis and progression of gallbladder adenocarcinoma. Our data may corroborate with the previous reports on other malignancies including prostate and renal cancers [7–11, 13, 29, 30]. Furthermore, this interpretation was strengthened by the prognostic data. In this study, we demonstrated that the survival time in patients with overexpression of PSCA and Oct-4 was significantly shorter than that in patients with lower expression. Multivariate Cox regression analysis indicated that overexpression of PSCA and Oct-4 was an independent predictor of worse prognosis in GBC. Taken together, PSCA and Oct-4 might be used as valuable biological markers to screen for neoplasia at a range of benign lesions and to reflect the prognosis of GBC. 

GBC is commonly diagnosed when it is unresectable, and palliative treatment is the main approach of medical care. However, the chemotherapy and radiation therapy offer little benefit in GBC [39]. Chemoresistance presents a major obstacle to the efficacy of chemotherapeutic treatment of cancer. Some studies demonstrated that chemoresistant cells displayed CSC features and overexpressed Oct-4 [27, 47, 48]. The regulatory mechanisms relating to Oct-4 in tumor chemoresistance have not been fully elucidated yet. Previous studies have revealed that expression of Oct-4 was increased in chemoresistant cancer cells due to DNA demethylation regulation of Oct-4. The overexpression of Oct-4 in liver cancer cells induced activation of TCL1, AKT, and ABCG2 to mediate chemoresistance [47]. In lung cancer-derived CD133-positive cells (LC-CD133 (+)), higher Oct-4 expression coexpressed the multiple drug-resistant marker ABCG2 and showed significant resistance to chemotherapy agents and radiotherapy. The treatment effect of chemoradiotherapy for LC-CD133 (+) could be improved by the treatment of Oct-4 siRNA [27]. Moreover, Hu et al. showed that murine Lewis lung carcinoma 3LL cells and human breast cancer MCF7 cells expressed Oct-4 at high levels. SiRNA against Oct-4 decreased the cancer stem cell-like cells (CSCLCs) number and markedly inhibited tumor growth. The Oct-4 might maintain the survival of CSCLCs partly through Oct-4/Tcl1/Akt1 by inhibiting apoptosis [48]. In our study, we found that 55.6% of gallbladder adenocarcinoma overexpressed Oct-4. Furthermore, Oct-4 was overexpressed in the poorly differentiated adenocarcinoma with larger tumor size (≥2 cm), having lymph node metastasis and invasion. The survival time in patients with overexpression of Oct-4 was significantly shorter than that in patients with lower Oct-4 expression. We speculated that overexpression of Oct-4 might play a crucial role in maintaining the self-renewing, cancer stem cell-like, chemoradioresistant properties and tumorigenesis in gallbladder adenocarcinoma. Overexpression of Oct-4 might be involved in chemotherapy and radiation therapy resistance in GBC by influencing CSC or CSCLCs. These data indicated that targeting Oct-4 may have important clinical applications in GBC therapy. Further studies are required to explore the unknown mechanisms relating to Oct-4 in GBC. 

To improve GBC patients' prognosis, there is an urgent need for developing novel therapeutic strategies. Recent advances in tumor biology have led to the development of specific molecular-targeted therapies. However, the current molecular-targeted therapeutic agents in GBC are limited. PSCA, a glycosylphosphatidylinositol- (GPI-) anchored cell-surface glycoprotein, has been reported to be overexpressed in more than 80% of prostate cancers [6, 8]. PSCA was also upregulated in some nonprostatic cancers including bladder, pancreatic, renal, gastric, and lung cancers [10–16, 49]. The significant cell-surface expression of PSCA in localized and advanced cancer, together with its restrictive expression in normal tissues, makes PSCA an attractive candidate target protein for metastatic and hormone-refractory prostate cancer [50–52]. Anti-PSCA monoclonal antibody treatment has been demonstrated to exhibit prostate cancer-specific cytotoxicity and to inhibit tumor growth and metastasis [50, 51]. It has also been reported that several PSCA-derived peptide vaccines could effectively induce PSCA-specific and long-lasting cellular and humoral immune responses against prostate cancer [53]. Related therapy studies of PSCA were also used to nonprostatic malignancies, including lung cancer [49] and pancreatic cancer [12]. Moreover, increasing researches showed that Oct-4 was also probably a potential gene therapeutic target for cancer, and targeting Oct-4 might have important clinical applications in cancer therapy. Knockdown of Oct-3/4 expression by RNA interference reduced migration and invasion of bladder cancer cells [28]. Based on these various research data, the PSCA and Oct-4 might be functionally important and novel therapeutic molecular targets for GBC. 

In conclusion, our study revealed that the expression of PSCA and Oct-4 was increased in gallbladder adenocarcinoma. The overexpression of PSCA and Oct-4 was correlated with decreased survival and might serve as important biological marker for reflecting the carcinogenesis, progression, metastasis, or invasive potential and prognosis of gallbladder carcinoma. Measurement of PSCA and Oct-4 expression could be a tool for early detection of GBC in benign lesions as well as population screening. The development of gene therapy to target PSCA and Oct-4 can be applied to GBC and may hold promise to improve patient survival. 

## Figures and Tables

**Table 1:** PSCA and Oct-4 expression in benign and malignant lesions of gallbladder.

Tissue types	Total no.	PSCA			Oct-4		
		Pos. nos. (%)	*χ* ^2^	*P	Pos. nos. (%)	*χ* ^2^	*P

GBC	108	59 (54.6)			60 (55.6)		
PT tissue	46	9 (19.6)	16.08	<0.01	10 (21.7)	14.89	<0.01
G. polyp	15	2 (13.3)	8.99	<0.01	2 (13.3)	9.39	<0.01
C. cholecystitis	35	5 (14.3)	17.40	<0.01	5 (14.3)	18.16	<0.01

*Compared with gallbladder adenocarcinoma (GBC). PT tissue: peritumoral tissue; G. polyp: gallbladder polyp; C. cholecystitis: chronic cholecystitis; Pos. nos. positive numbers.

**Table 2:** The association of PSCA and Oct-4 expression with the clinicopathological characteristics of gallbladder adenocarcinoma.

Clinicopathological characteristics	Total no.	PSCA			Oct-4		
		Pos. nos. (%)	χ^2^	*P*	Pos. nos. (%)	*χ* ^2^	*P*
Sex							
Male	31	16 (51.6)	0.16	>0.05	17 (54.8)	0.01	>0.05
Female	77	43 (55.8)			43 (55.8)		
Age (years)							
≤45	24	14 (58.3)	0.17	>0.05	16 (66.7)	1.54	>0.05
>45	84	45 (53.6)			44 (52.4)		
Differentiation*							
Well	36	14 (38.8)	15.12	<0.01	13 (36.1)	13.31	<0.05
Moderately	31	16 (51.6)			18 (58.1)		
Poorly	30	25 (83.3)			24 (80.0)		
Mucinous A.	11	4 (36.4)			5 (45.5)		
Tumor size (cm)							
<2 cm	31	12 (38.7)	4.47	<0.05	10 (32.3)	9.56	<0.01
≥2 cm	77	47 (61.0)			50 (64.9)		
Lymph node metastasis							
No	49	19 (38.8)	8.04	<0.01	19 (38.8)	10.23	<0.01
Yes	59	40 (67.8)			41 (69.5)		
T stages							
T1	14	5 (35.7)	7.73	<0.05	4 (28.6)	17.24	<0.01
T2	35	15 (42.9)			13 (37.1)		
T3	37	23 (62.2)			25 (67.6)		
T4	22	16 (72.7)			18 (81.8)		
Gallstones							
No	50	27 (54.0)	0.01	>0.05	28 (56.0)	0.01	>0.05
Yes	58	32 (55.2)			32 (55.2)		

*Comparison between well-differentiated and moderately differentiated adenocarcinomas: *χ*_PSCA_^2^ = 1.09, *P* > 0.05; *χ*_Oct-4_^2^ = 3.23, *P* > 0.05.

*Comparison between wel-differentiated and poorly differentiated adenocarcinomas: *χ*_PSCA_^2^ = 7.29, *P* < 0.01; *χ*_Oct-4_^2^ = 6.98, *P* < 0.01.

*Comparison between moderately differentiated and poorly differentiated adenocarcinoma: *χ*_PSCA_^2^ = 6.96, *P* < 0.01; *χ*_Oct-4_^2^ = 3.42, *P* < 0.05.

Mucinous A.: mucinous adenocarcinoma.

**Table 3:** Relationship between PSCA and Oct-4 expression, clinicopathological characteristics, and average survival of gallbladder adenocarcinoma patients.

Clinicopathological characteristics	Samples (*n*)	Average survival (month)	*P* value
Sex			
Male	19	10.0 (4–16)	0.910
Female	48	10.0 (4–18)	
Age (years)			
≤45	11	8.0 (4–14)	0.121
>45	56	10.0 (4–18)	
Pathological types			
Adenoma	8	12.0 (8–18)	0.031
Well	20	10.0 (4–18)	
Moderately	20	10.0 (4–18)	
Poorly	12	8.0 (4–10)	
Mucinous A.	7	10.0 (6–16)	
Tumor size (cm)			
<2	20	14.0 (4–18)	0.003
≥2	47	8.0 (4–18)	
Lymph node metastasis			
No	36	12.0 (4–18)	0.005
Yes	31	8.0 (4–18)	
T stages			
T1	10	12.6 (6–18)	0.003
T2	26	11.4 (4–18)	
T3	23	9.5 (4–18)	
T4	8	7.0 (4–10)	
Operative procedure			
Radical	26	12.7 (4–18)	<0.001
Nonradical	41	8.9 (4–16)	
PSCA			
+	35	9.3 (4–18)	0.013
−	32	11.6 (4–18)	
Oct-4			
+	37	9.5 (4–18)	0.029
−	30	11.5 (4–18)	
PSCA + Oct-4			
PSCA (+) Oct-4 (+)	24	8.9 (4–18)	0.021
PSCA (+) Oct-4 (−)	11	10.7 (4–16)	
PSCA (−) Oct-4 (+)	13	9.7 (4–16)	
PSCA (−) Oct-4 (−)	19	12.5 (6–18)	

**Table 4:** Multivariate Cox regression analysis of overall survival in 67 gallbladder carcinoma patients.

Groups	Factors	RC*	SE*	RR*	*P* value	95% confidence interval	
						Lower	Upper
Pathological types	Adenoma /well/moderately /*poorly*/mucinous adenocarcinoma	0.589	0.425	1.83	0.305	0.78	4.1
Tumor size (cm)	<2.0/≥2.0	0.978	0.433	3.01	0.021	1.15	6.27
Lymph node metastasis	No/yes	1.114	0.521	3.33	0.041	1.10	8.46
T stages	T1/T2/T3/T4	0.978	0.318	2.64	0.021	1.49	4.85
Resection procedure	Radical/nonradical	1.409	0.515	4.09	0.005	1.52	11.08
PSCA	−/+	1.025	0.487	3.36	0.045	1.07	7.24
Oct-4	−/+	0.754	0.336	2.37	0.039	1.10	4.11

*RR: relative risk; *SE: standard error; *RC: regression coefficients.
